# Mesophyll conductance in cotton bracts: anatomically determined internal CO_2_ diffusion constraints on photosynthesis

**DOI:** 10.1093/jxb/ery296

**Published:** 2018-08-14

**Authors:** Jimei Han, Zhangying Lei, Jaume Flexas, Yujie Zhang, Marc Carriquí, Wangfeng Zhang, Yali Zhang

**Affiliations:** 1 The Key Laboratory of Oasis Eco-agriculture, Xinjiang Production and Construction Group, Shihezi University, Shihezi, P.R. China; 2 Research Group in Plant Biology under Mediterranean Conditions, Universitat de les Illes Balears-Instituto de Agroecología y Economía del Agua (INAGEA), Palma, Illes Balears, Spain

**Keywords:** Anatomical structures, CO_2_ diffusion, cotton bracts, mesophyll conductance, non-leaf green organs, stomatal conductance

## Abstract

Mesophyll conductance (*g*_m_) has been shown to affect photosynthetic capacity and thus the estimates of terrestrial carbon balance. While there have been some attempts to model *g*_m_ at the leaf and larger scales, the potential contribution of *g*_m_ to the photosynthesis of non-leaf green organs has not been studied. Here, we investigated the influence of *g*_m_ on photosynthesis of cotton bracts and how it in turn is influenced by anatomical structures, by comparing leaf palisade and spongy mesophyll with bract tissue. Our results showed that photosynthetic capacity in bracts is much lower than in leaves, and that *g*_m_ is a limiting factor for bract photosynthesis to a similar extent to stomatal conductance. Bract and the spongy tissue of leaves have lower mesophyll conductance than leaf palisade tissue due to the greater volume fraction of intercellular air spaces, smaller chloroplasts, lower surface area of mesophyll cells and chloroplasts exposed to leaf intercellular air spaces and, perhaps, lower membrane permeability. Comparing bracts with leaf spongy tissue, although bracts have a larger cell wall thickness, they have a similar *g*_m_ estimated from anatomical characteristics, likely due to the cumulative compensatory effects of subtle differences in each subcellular component, especially chloroplast traits. These results provide the first evidence for anatomical constraints on *g*_m_ and photosynthesis in non-leaf green organs.

## Teaser

Photosynthetic capacity in bracts is much lower than in leaves, and mesophyll conductance is a limiting factor for bract photosynthesis.

## Introduction

To reach the sites of carboxylation within chloroplasts of leaves of C_3_ plants, CO_2_ must diffuse through stomata and mesophyll. Stomatal CO_2_ diffusion occurs from the ambient air just outside the leaf to the substomatal cavities, while mesophyll CO_2_ diffusion occurs from the substomatal cavities to just outside the mesophyll cell wall (i.e. gas phase resistance) and to all the cell structures (cell wall, plasma membrane, cytoplasm, chloroplast envelope membranes, and stroma) that CO_2_ must necessarily pass through to reach the carboxylation center (i.e. liquid phase resistances; Evans *et al.*, 1994, 2009). Although CO_2_ diffusion through the leaf has been widely studied, this fairly complex process is not fully understood yet (Evans *et al.*, 2009; Flexas *et al.*, 2012; Tosens *et al.*, 2012*b*). Many studies have shown that mesophyll conductance (*g*_m_) significantly limits photosynthesis and often can be the main limitation to photosynthesis (Flexas *et al.*, 2008; Tosens *et al.*, 2012b;Galmés *et al.*, 2014; Peguero-Pina *et al.*, 2017). In the gas phase conductance, CO_2_ diffusion through intercellular air spaces may be hindered by leaf thickness, mesophyll cell shape, relative distribution of palisade and spongy tissue (Evans *et al.*, 2009), and the volume fraction of intercellular air spaces (*f*_ias_) (Syvertsen *et al.*, 1995; Terashima *et al.*, 1995). Regarding the liquid phase conductance, it is mainly constrained by the cell wall thickness (*T*_cw_), the chloroplast dimensions, and the mesophyll and chloroplast surface area exposed to leaf intercellular air spaces (*S*_m_/*S* and *S*_c_/*S*) (Evans *et al.*, 1994, 2009; Tosens *et al.*, 2012*b*; Tomás *et al.*, 2013). These anatomical structures have been observed to strongly differ between different species (Tomás *et al.*, 2013; Peguero-Pina *et al.*, 2016) or even within the same species growing under complex and variable growth environments (Terashima *et al.*, 2011; Tosens *et al.*, 2012*a*).

Mesophyll conductance (*g*_m_) is important in setting the plant photosynthetic capacity. However, the neglect of CO_2_ drawdown from the substomatal cavities to chloroplasts in the photosynthetic model at the leaf level (Niinemets, 2007) and global carbon cycle model (Sun *et al.*, 2014), by using intercellular CO_2_ concentration (*C*_i_) instead of chloroplastic CO_2_ concentration (*C*_c_), results in an underestimation of the biochemical parameters, particularly the maximum carboxylation rate (*V*_cmax_) and maximum electron transport rate (*J*_max_). To avoid such underestimation, some modeling studies have focused on *g*_m_ and estimated photosynthetic parameters using *C*_c_ at the leaf (Ethier and Livingston, 2004; Sharkey *et al.*, 2007; Gu *et al.*, 2010; Sharkey, 2016) and whole canopy scales (Sun *et al.*, 2014).

It has been shown that non-leaf green organs are also an important source of assimilated carbon at the ecological and agricultural scales (Tambussi *et al.*, 2007; Redondo-Gómez *et al.*, 2010; Pengelly *et al.*, 2011; Hu *et al.*, 2012; Jia *et al.*, 2015; Zhang *et al.*, 2015), and thus make a considerable contribution to the terrestrial carbon exchange. However, the importance of the mesophyll diffusion limitation for photosynthesis has yet to be studied in the non-leaf green organs.

**Fig. 1.**
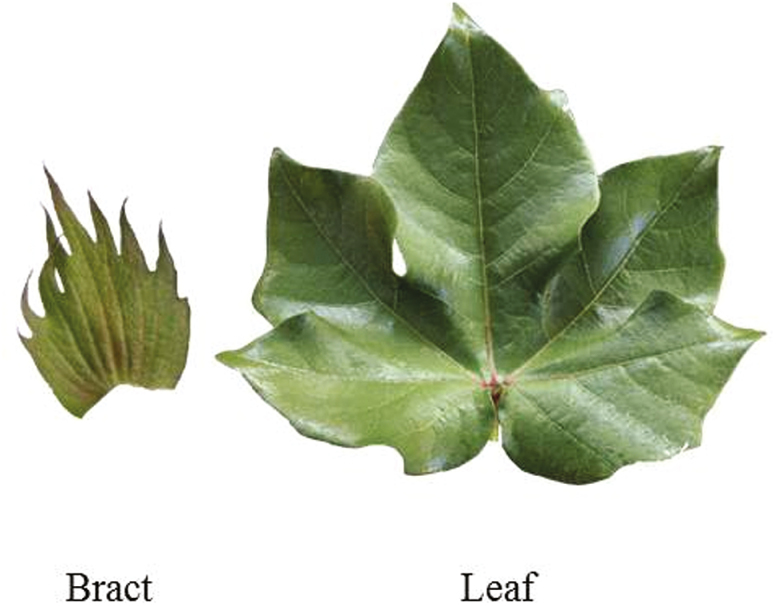
A bract and a leaf from the cotton plant.

Currently, in agricultural production, it has been demonstrated that cotton bracts, non-leaf green organs that cover cotton fruits, make a significant contribution to cotton carbon gain especially in the later growth stages (Hu *et al.*, 2012). A higher water use efficiency (Hu *et al.*, 2013) and drought tolerance (Zhang *et al.*, 2015) in bracts than in leaves was also reported. However, no research has focused on the relationship between these photosynthetic characteristics and the property of CO_2_ diffusion, especially mesophyll CO_2_ diffusion in the bracts. Without explicit consideration of *g*_m_, some photosynthetic parameters in bracts would have been underestimated just like in the leaves. Generally, the difference in morphological and anatomical structures between leaves and bracts is obvious (Fig. 1; Hu *et al.*, 2012). But the difference in internal mesophyll structure is still not clear. Research has shown that in the cotton bracts there is only one type of photosynthetic tissue, which is similar to the spongy tissue of the leaf. However, there are smaller and less numerous chloroplasts and more loose tissue in the spongy tissue, from which we speculate there is a larger mesophyll limitation in the bract than in the leaf. To the best of our knowledge, no previous study has analysed the effect of internal structures of palisade and spongy tissues on mesophyll diffusion of CO_2_. To fill this gap, cotton leaves and bracts were studied and we compared the anatomy of palisade and spongy tissue structures with that of bracts. The aims of the study were (i) to determine if bracts are constitutively more limited than leaves for CO_2_ diffusion; (ii) to reveal if the different types of tissues lead to a difference in mesophyll diffusion between the leaf and the bract; and (iii) to quantify the contribution of mesophyll structures to setting differences in *g*_m_ and photosynthesis between leaves and bracts.

## Materials and methods

### Plant material

Cotton (*Gossypium hirsutum* L. ‘Xinluzao 45’) plants were grown at an experimental field of Shihezi Agricultural College, Shihezi University, Xinjiang, China (45°19′N, 86°03′E). Before sowing, drip irrigation tubes were installed beneath the plastic film, which supplied water for the cotton. Seeds were sown on 21 April 2015 in rows 12 cm apart at a plant density of 1.8 × 10^5^ ha^−1^. The plots were fertilized before sowing with 240 kg N ha^−1^ (urea), 170 kg P_2_O_5_ ha^−1^ [(NH_4_)_3_PO_4_], and 1500 kg ha^−1^ organic fertilizer (235 g kg^−1^ organic matter, 18 g kg^−1^ total N, 14 g kg^−1^ total P, and 22 g kg^−1^ total K). An additional 120 kg N ha^−1^ (urea) was applied by drip irrigation during the growing seasons. Weeds and pests were controlled in the field using standard management practices. At peak bolling stage (100–110 days after sowing), the topmost fully expanded leaf on the main stem and bract on the fruit branch of the cotton were selected for the experiment. Meteorological data during the growing season are shown in Supplementary Fig. S1 at *JXB* online.

### Gas exchange and chlorophyll fluorescence

**Fig. 2.**
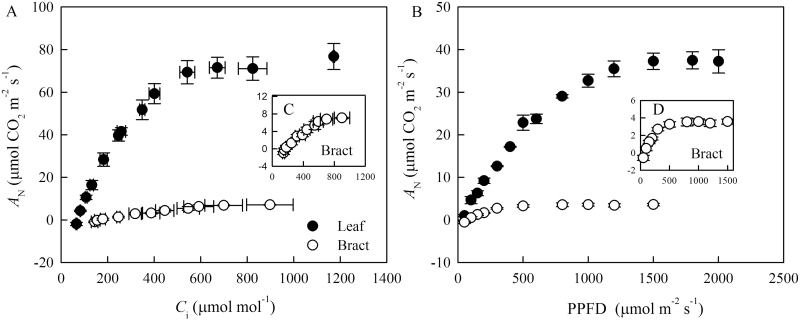
Net CO_2_ assimilation rate (*A*_N_) expressed on the basis of leaf area as a function of intercellular CO_2_ concentration (*C*_i_) (A) and photosynthetically active photon flux density (PPFD) (B) in cotton leaves and bracts. Bracts data are also shown in the insets (C, D). Values are means±SE.

Gas exchange and chlorophyll fluorescence were measured simultaneously on the main leaves and bracts, using an open gas-exchange system (Li-6400; Li-Cor, Inc., Lincoln, NE, USA) connected to a leaf fluorometer chamber (Li-6400-40; Li-Cor Inc.). The bolls were detached from the bracts so as to be able to clamp the bract to obtain the CO_2_ and light response curves. Leaf temperature was set to 30 °C. The vapor pressure deficit (VPD) was between 2 and 3 kPa and the flow rate was set at 300 μmol s^−1^. The ratio of red:blue light was set to 90:10% PPFD to maximize stomatal aperture. CO_2_ concentration in the Li-6400 leaf chamber was provided by a CO_2_ cylinder and maintained constant at 400 μmol CO_2_ mol^−1^. Light-response curves were obtained under the light intensities 2000, 1800, 1500, 1200, 1000, 800, 500, 300, 200, 150, 100, 50, and 0 μmol m^−2^ s^−1^ for leaves and 1500, 1200, 1000, 800, 500, 300, 200, 150, 100, 50, and 0 μmol m^−2^ s^−1^ for bracts. CO_2_-response curves in light saturating conditions were obtained by first determining the parameters at 2000 μmol m^−2^ s^−1^ photosynthetically active photon flux density (PPFD) for leaves and at 1000 μmol m^−2^ s^−1^ for bracts (see Fig. 2B for *A*_N_–PAR curves confirming light saturating conditions for both leaves and bracts). Photosynthesis was induced with an ambient CO_2_ concentrations (*C*_a_) of 400 μmol mol^−1^ and 21% O_2_ surrounding the leaf. Once steady state was reached (usually 20 min after clamping the leaf), data were recorded. Immediately after, the air inlet pipe was connected to a 2% O_2_ and 98% N_2_ medical gas bag, and a CO_2_-response curve (net assimilation rate (*A*_N_)–*C*_i_ curve) was obtained. After that, the Li-COR inlet was disconnected from N_2_ medical gas bag (i.e. air with 21% O_2_ was supplied again to the plant). After reaching steady state, another *A*_N_–*C*_i_ curve was obtained. In regard to the *A*_N_–*C*_i_ curve, gas exchange and chlorophyll fluorescence were first measured at *C*_a_ of 400 μmol mol^−1^; then *C*_a_ was decreased stepwise to 50 μmol mol^−1^. Upon completion of measurements at low *C*_a_, *C*_a_ was returned to 400 μmol mol^−1^ to restore the original *A*_N_. Then *C*_a_ was increased stepwise to complete the curve. The number of different *C*_a_ values used for the curves was 12, and the time interval between two consecutive measurements at different *C*_a_ was restricted to 2–4 min, so that each curve was completed in 30–50 min. The actual photochemical efficiency of photosystem II (Φ_PSII_) was determined by measuring steady state fluorescence (*F*_s_) and maximum fluorescence during a light-saturating pulse of ca. 8000 μmol m^−2^ s^−1^ (*F*_m_′):

$$\Phi_{\text{PSII}}=\frac{F_{\text{m}}\prime -F_{\text{s}}}{F_{\text{m}}\prime }$$ (1)

The electron transport rate (*J*_flu_) was then calculated as:

$$J_{\text{flu}}=\Phi_{\text{PSII}}\times \text{PPFD}\times \alpha \times \beta$$ (2)

Where PPFD is the photosynthetically active photon flux density, α is leaf absorptance and β reflects the partitioning of absorbed quanta between photosystems II and I (PSI and PSII). α was assumed to be 0.85 and β to be 0.5. Because numerous studies have shown that the estimation of *J*_flu_ is affected by PSI and the signal-to-noise ratio in the determination of *F*_m_′ at high light, the electron transport rate from gas exchange under 2% O_2_ conditions (*J*_A_) was used to calibrate *J*_flu_ (see Pons *et al.*, 2009 for details).

*g*_m_ was estimated by the variable *J* method (Harley *et al.*, 1992*a*) as:

$$g_{\text{m}(\text{Harley)}}=\frac{A_{\text{N}}}{C_{\text{i}}-\frac{\Gamma *\times [J_{\text{flu}}+8\left(A_{\text{N}}+R_{\text{d}}\right)]}{J_{\text{flu}}-4\left(A_{\text{N}}+R_{\text{d}}\right)}}$$ (3)

where Γ* is the CO_2_ compensation point in the absence of mitochondrial respiration and *R*_d_ is day respiration. *A*_N_ and *C*_i_ were taken from gas-exchange measurements at saturating light and the value of Γ* (44.04) at 30 °C from Bernacchi *et al.* (2002) used for the variable *J* methods of calculating *g*_m_:

$$\Gamma^{\text{*}}=\text{exp}(13.49-\frac{24460}{8.314\times \left(273.15+T_{\text{L}}\right)})$$

where *T*_L_ is the leaf temperature (°C). *R*_d_ was assumed to be half of the measured dark respiration (*R*_n_, *R*_d_=*R*_n_/2) (Villar *et al.*, 1995; Niinemets *et al.*, 2005). *R*_n_ was determined by gas exchange (Li-6400), after plants had been dark-adapted for more than half an hour in the evening. CO_2_ leakage of the leaf cuvette was determined by performing *A*_N_–*C*_i_ response curves with photosynthetically inactive leaves and bracts enclosed in the leaf chamber (obtained by heating the leaves until no variable chlorophyll fluorescence was observed), and used to correct measured leaf fluxes (Flexas *et al.*, 2007).

### Estimation of *g*_m_ by *A*_N_–*C*_i_ curve fitting

The curve-fitting method introduced by Sharkey (2016) was used to obtain an alternative estimate of *g*_m_. This method is based on changes in the curvature of *A*_N_–*C*_i_ response curves due to a finite *g*_m_. By non-linear curve fitting minimizing the sum of squared model deviations from the data, *g*_m_ can be estimated from observed data. The same data were used for estimation of *g*_m_ by the methods of Sharkey (2016) and Harley *et al.* (1992*a*).

### Estimation of *V*_cmax_ and *J*_max_

The *A*_N_–*C*_c_ curves were fitted based upon the model of Farquhar *et al.* (1980), which was later modified and developed by Harley *et al.* (1992*a*,b). According to the biochemical model, *A*_N_ can be expressed as:

$$A_{\text{N}}=V_{\text{c}}-0.5V_{\text{o}}-R_{\text{d}}=\text{min}\{A_{\text{c}},A_{\text{j}},A_{\text{p}}\}$$ (4)

With

$$A_{\text{c}}=\frac{V_{\text{cmax}}\times (C_{\text{c}}-\Gamma^{*})}{C_{\text{c}}+K_{\text{c}}\times (1+\frac{\text{O}}{K_{\text{o}}})}-R_{\text{d}}$$ (5)

$$A_{\text{j}}=\frac{J_{\text{max}}\times (C_{\text{c}}-\Gamma^{*})}{4C_{\text{c}}+8\Gamma^{*}}-R_{\text{d}}$$ (6)

where *A*_c_, *A*_j_, and *A*_p_ are the net CO_2_ assimilation rate limited by Rubisco, ribulose 1,5-bisphosphate (RuBP), and triose phosphate use (TPU), respectively. *V*_c_ and *V*_o_ are rates of carboxylation and oxygenation of Rubisco. O is the O_2_ concentration at the sites of carboxylation within chloroplasts. *K*_c_ and *K*_o_ are Michaelis–Menten constants for carboxylation and oxygenation, respectively (Bernacchi *et al.*, 2002). Best-fit values of the parameters *J*_max_ and *V*_cmax_ were obtained using the whole curve data points (i.e. Eqn 4) rather than a portion of the curve according to ‘method I’ of Miao *et al.* (2009).

### Electron microscopy

Leaf and bract samples (4 mm×1.5 mm) were fixed by infiltration of 2.5% glutaraldehyde and 3% paraformaldehyde in 0.1 mol l^−1^ phosphate buffer (pH 7.2) under vacuum. Leaf samples were fixed again in 1% osmium tetroxide overnight and dehydrated in a graded acetone series and embedded in Spurr’s resin. Semi-thin leaf cross-sections of 4 μm for light microscopy and ultra-thin (80 nm) cross-sections were prepared with an ultramicrotome (Leica Ultracut, Germany). The sections for light microscopy were stained with toluidine blue. Ultra-thin cross-sections for transmission electron microscopy were stained with uranyl acetate and lead citrate double staining, observed under an electron microscope (TEM HT7700, Japan), and electron micrographs were taken with a digital camera (BH-2, Olympus). Each anatomical trait per replicate was measured 6–10 times. It should be noted that electron micrographs of palisade and spongy tissues were taken and then quantified according to the below methods and formulas.

The surface of mesophyll cells and chloroplasts exposed to leaf intercellular air spaces (*S*_m_/*S* and *S*_c_/*S*) were calculated following the method of Syvertsen *et al.* (1995) as:

$$\frac{S_{\text{m}}}{S}\;=\frac{L_{\text{mes}}\times F}{W}$$ (7)

where *L*_mes_ is the total length of mesophyll cells facing the intercellular air space in the palisade tissue or spongy tissue section, *F* is the curvature correction factor that depends on the shape of the cells (Thain, 1983; Evans *et al.*, 1994), and *W* is the width of the section measured.

$$\frac{S_{\text{c}}}{S}\;=\frac{L_{\text{c}}\times F}{W}$$ (8)

where *L*_c_ is the total length of chloroplast surface area facing the intercellular air space in the palisade tissue or spongy tissue sections.

The volume fraction of intercellular air space (*f*_ias_) was determined as:

$$f_{\text{ias}}=1-\frac{\sum^{\text{​}}S_{\text{s}}}{t_{\text{mes}}\times W}$$ (9)

where *t*_mes_ is the mesophyll thickness between the two epidermal layers and ∑*S*_s_ is the sum of the cross-sectional areas of mesophyll cells.

The volume fraction of intercellular air space of palisade tissue and spongy tissue was determined respectively as:

$$f_{\text{ias}}(\text{palisade})=1-\frac{\sum^{\text{​}}S_{\text{pal}}}{t_{\text{pal}}\times W}$$ (10)

$$f_{\text{ias}}(\text{spongy})=1-\frac{\sum^{\text{​}}S_{s\text{po}}}{t_{\text{spo}}\times W}$$ (11)

Where ∑*S*_pal_ is the sum of the cross-sectional areas of palisade tissue cells, *t*_pal_ is the palisade tissue thickness, ∑*S*_spo_ is the sum of the cross-sectional areas of spongy tissue cells, *t*_spo_ is the spongy tissue thickness.

Chloroplast length (*L*_chl_), chloroplast thickness (*T*_chl_) and *T*_cw_ were obtained at different positions in each sample at ×30000 magnifications. For a given section, all characteristics were determined in at least three different fields of view, and at least three different sections were analysed.

The cross-section of a chloroplast is assumed to be oval. Therefore, the cross-section area of chloroplast (Area_chl_) was calculated in the palisade tissue or spongy tissue section as:

$${\text{Area}}_{\text{chl}}=\pi \times L_{\text{chl}}\times T_{\text{chl}}$$ (12)

where π is the ratio of the circumference of a circle to its diameter.

### *g*_m_ modeled from anatomical characteristics

According to the quantitative one-dimensional gas diffusion model of Niinemets and Reichstein (2003) further used by Tosens *et al.* (2016), mesophyll conductance of total leaf, palisade, spongy tissue and bract was estimated using the leaf anatomical characteristics (i.e. *g*_m(anatomy)_). In the model, *g*_m(anatomy)_ is separated into gas phase conductance and liquid phase conductance (Evans *et al.*, 1994):

$$g_{\text{m(anatomy)}}=\frac{1}{\frac{1}{g_{\text{ias}}}+\frac{R\times T_{\text{K}}}{H\times g_{\text{liq}}}}$$ (13)

where *g*_ias_ is conductance from substomatal cavities to outer surface of cell walls and *g*_liq_ is the conductance from outer surface of cell walls to chloroplasts; *R* is the gas constant (Pa m^3^ K^−1^ mol^−1^), *H* is the Henry’s law constant (Pa m^3^ mol^−1^), and *T*_k_ is the absolute temperature (K). *H*/(*R*×*T*_k_) is needed to convert *g*_liq_ to a gas phase equivalent conductance (Niinemets and Reichstein, 2003).

The gas phase conductance (*g*_ias_) was calculated as described in Niinemets and Reichstein (2003):

$$g_{\text{ias}}=\frac{D_{\text{a}}\times f_{\text{ias}}}{\Delta L_{\text{ias}}\times \varsigma }$$ (14)

where Δ*L*_ias_ was taken as half the mesophyll thickness (Niinemets and Reichstein, 2003), *D*_a_ (m^−2^s^−1^) is the diffusion coefficient for CO_2_ in the gas phase (1.51 × 10^–5^ m^−2^ s^−1^ at 25 °C), and ς is the diffusion path tortuosity (m m^−1^) for which we used a default value of 1.57 m m^−1^ (Syvertsen *et al.*, 1995; Niinemets and Reichstein, 2003).

The total liquid phase conductance is provided by the sum of the inverse of serial conductances (Tosens *et al.*, 2016):

$$\frac{1}{g_{\text{liq}}}=(\frac{1}{g_{\text{cw}}}+\frac{1}{g_{\text{pl}}}+\frac{1}{g_{\text{ct}}}+\frac{1}{g_{\text{en}}}+\frac{1}{g_{\text{st}}})\times \frac{S_{\text{c}}}{S}$$ (15)

where the partial conductances are those for cell wall (*g*_cw_), plasmalemma (*g*_pl_), cytosol (*g*_ct_), chloroplast envelope (*g*_en_), and chloroplast stroma (*g*_st_). *g*_cw_, *g*_ct_, and *g*_st_ were calculated as described in Tomás *et al.* (2013). Cell wall porosity (*p*_cw_) varied with *T*_cw_ according to Tosens *et al.* (2016) (*p*_cw_=−0.3733×*T*_cw_+0.3378). We used an estimate of 0.0035 m s^−1^ for *g*_pl_ and *g*_en_ (Tosens *et al.*, 2012*b*). Conductance in units of m s^−1^ can be converted into molar units considering that:

$$g[{\text{mol m }}^{-2}{\text{ s}}^{-1}]=g[\text{m}\;{\text{s}}^{-1}]\times 44.6\times \frac{273.15}{273.15+T_{\text{L}}}\times \frac{P}{101.325}$$

where *T*_L_ is the leaf temperature (°C) and *P* (Pa) is the air pressure.

### Quantitative analysis of partial limitation of *g*_m_ modeled

According to Tosens *et al.* (2016), the limitations derived from different components were calculated as:

$$L_{\text{ias}}=\frac{g_{\text{m-anatomy}}}{g_{\text{ias}}}$$ (16)

$$L_{\text{i}}=\frac{g_{\text{m(anatomy)}}}{g_{\text{i}}\times \frac{S_{\text{c}}}{S}}$$ (17)

where *g*_m(anatomy)_ is mesophyll conductance estimated from anatomical characteristics applying the model of Niinemets and Reichstein (2003) as modified by Tosens *et al.* (2016), *L*_ias_ is the limitation derived from the gas phase component, *L*_i_ is the component limitation in the cell wall, plasmalemma, cytoplasm, chloroplast envelope, and stroma, *g*_i_ refers to the component diffusion conductance of the corresponding diffusion pathways.

### Relative limitation analyses on *A*_N_

The relative limitation on *A*_N_ was analysed in cotton leaves and bracts. According to Grassi and Magnani (2005), relative stomatal limitation (*l*_s_), mesophyll limitation (*l*_m_), and biochemical limitation (*l*_b_) were investigated in the cotton leaves and bracts. *l*_m_ was calculated using *g*_m_ calculated from gas-exchange plus fluorescence measurements following Harley *et al.* (1992*a*) (*g*_m(Harley)_), from anatomical characteristics applying the model of Niinemets and Reichstein (2003) as modified by Tosens *et al.* (2016) (*g*_m(anatomy)_) and from the average value between the anatomy and Harley methods. The relative changes in light- saturated assimilation can be expressed in terms of parallel relative changes in stomatal and mesophyll conductance and in biochemical capacity as follows:

$$\frac{\text{d}A_{\text{N}}}{A_{\text{N}}}=\text{SL}+\text{MCL}+\text{BL}=l_{\text{s}}\times \frac{\text{d}g_{\text{s }}}{g_{\text{s }}}+l_{\text{m}}\times \frac{\text{d}g_{\text{m}}}{g_{\text{m}}}+l_{\text{b}}\times \frac{\text{d}v_{\text{cmax}}}{v_{\text{cmax}}}$$ (18)

$$l_{\text{s}}=\frac{\frac{g_{\text{tot }}}{g_{\text{s }}}\times \frac{\partial A_{\text{N}}}{\partial C_{\text{c}}}}{g_{\text{tot }}+\frac{\partial A_{\text{N}}}{\partial C_{\text{c}}}}$$ (19)

$$l_{\text{m}}=\frac{\frac{g_{\text{tot }}}{g_{\text{m}}}\times \frac{\partial A_{\text{N}}}{\partial C_{\text{c}}}}{g_{\text{tot }}+\frac{\partial A_{\text{N}}}{\partial C_{\text{c}}}}$$ (20)

$$l_{\text{b}}=\frac{g_{\text{tot }}}{g_{\text{tot }}+\frac{\partial A_{\text{N}}}{\partial C_{\text{c}}}}$$ (21)

where *g*_tot_ is total conductance to CO_2_ between the leaf surface and the sites of carboxylation (1/*g*_tot_=1/*g*_s_+1/*g*_m_); *l*_s_, *l*_m_, and *l*_b_ are the corresponding relative limitation (0<*l*_*i*_<1, *i*=s, m, b). Here, $\frac{\partial A_{\text{N}}}{\partial C_{\text{c}}}$ was calculated as the slope of *A*_N_–*C*_c_ response curves over a *C*_c_ range of 50–100 µmol mol^–1^ (Tomás *et al.*, 2013)

### Chlorophyll content, mass per area and nitrogen content

The chlorophyll content of leaves and bracts was determined in eight leaf discs (0.186 cm^2^ each). Discs of the green organs were extracted in 80% (v/v) acetone for 24 h at room temperature in the dark. The absorbance of an extract was measured with a spectrophotometer, and the chlorophyll content was calculated according to Lichtenthaler (1987).

Leaf mass per unit area (LMA) is the ratio of dry weight and leaf area. Dry weight was determined from oven-dried certain area of leaf discs after 48 h at ca. 80 °C. Leaf density was defined as LMA divided by leaf thickness.

For the measurement of nitrogen content, leaves and bracts were harvested on the same day. Total nitrogen content of the dried tissues was determined according to the micro-Kjeldahl method (Schuman *et al.*, 1972).

### Statistical analysis

Statistical analysis was performed with SPSS 17.0 for Windows (SPSS Inc., Chicago, IL, USA). All data were tested by analysis of variance (ANOVA). The significance of differences between treatment means was determined by the Student–Newman–Keuls (S-N-K) test at the 0.05 probability level. Data are presented as the means±standard error (SE) of three replicates.

## Results

### Difference in photosynthetic properties between cotton leaves and bracts

**Fig. 3.**
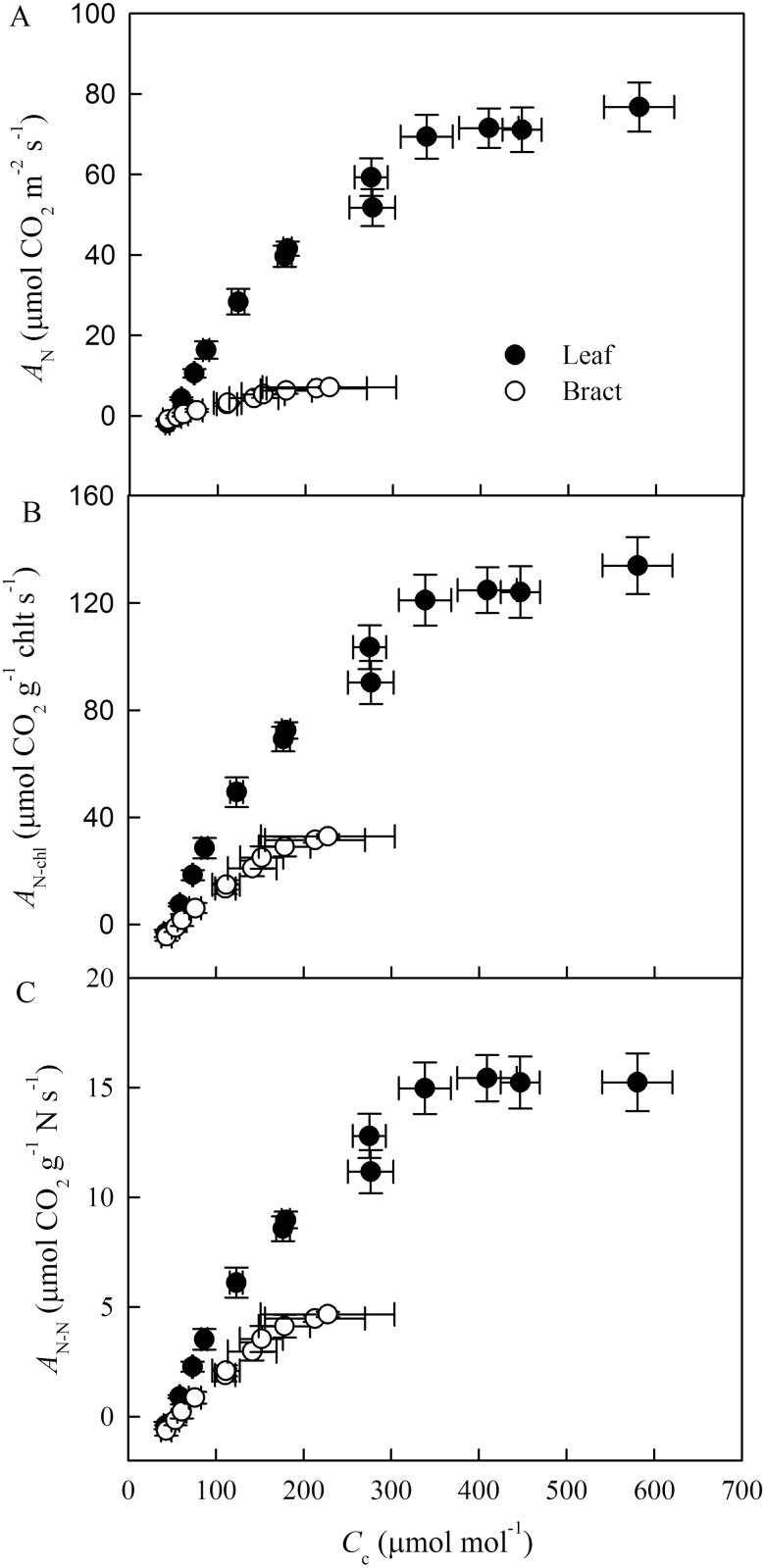
Net CO_2_ assimilation rate expressed on the basis of area (*A*_N_) (A), chlorophyll (*a*+*b*) (*A*_N-chl_) (B), and nitrogen content (*A*_N-N_) (C) as a function of chloroplastic CO_2_ concentration (*C*_c_) in cotton leaves and bracts. Values are means±SE.

The net CO_2_ assimilation rate (*A*_N_), stomatal conductance (*g*_s_), and mesophyll conductance (*g*_m_) were significantly higher in leaves than in cotton bracts (Table 1). In cotton leaves the *A*_N_ response to increasing *C*_i_ initially increased, then peaked, and finally remained stable above 600 μmol mol^−1^*C*_i_. *A*_N_ as a function of *C*_i_ in cotton bracts was lower than that in leaves (Fig. 2A). Relative to leaves, *A*_N_ response to increasing PPFD in the bracts was minor and saturated at a lower irradiance (Fig. 2B). Both chlorophyll (*a*+*b*) content and the ratio between chlorophyll *a* and chlorophyll *b* (Chl*a*/*b*) of cotton bracts were much lower than those in the cotton leaves (Table 2). The nitrogen content of bracts was 21% lower than that of leaves (Table 2). Larger *V*_cmax_ and *J*_max_ derived from *A*_N_–*C*_c_ curves were observed in the leaf than in the bract. The electron transport rate from chlorophyll fluorescence (*J*_flu_) calibrated by electron transport from gas exchange (*J*_A_) under 2% O_2_ conditions was close to *J*_max_ based on the *C*_c_. There was a difference in *A*_N_, *A*_N-chl_, and *A*_N-N_ as a function of *C*_c_ between leaves and bracts (Fig. 3A–C).

**Table 1.** Net assimilation rate (*A*_N_), stomatal conductance (*g*_s_), and mesophyll conductance (*g*_m_) estimated by three independent methods: using gas-exchange plus fluorescence measurements following Harley *et al.* (1992*a*), the curve-fitting method of Sharkey (2016), and using *g*_m_ estimated from anatomical characteristics applying the model of Niinemets and Reichstein (2003) as modified by Tosens *et al.* (2016) in total leaves, palisade and spongy tissue of leaves, and bracts

		*A*_N_ (µmol CO_2_ m^−2^ s^−1^)	*g*_s_ (mol H_2_O m^−2^ s^−1^)	*g*_m(Harley)_ (mol CO_2_ m^−2^ s^−1^)	*g*_m(Sharkey)_ (mol CO_2_ m^−2^ s^−1^)	*g*_m(anatomy)_ (mol CO_2_ m^−2^ s^−1^)
Leaf	Total	37.26 ± 1.93a	0.62 ± 0.07a	0.37 ± 0.01a	0.48 ± 0.10a	0.33 ± 0.03a
	Palisade tissue	**—**	**—**	**—**	**—**	0.25 ± 0.02b
	Spongy tissue	**—**	**—**	**—**	**—**	0.14 ± 0.02c
Bract	Total	3.58 ± 0.28b	0.06 ± 0.00b	0.03 ± 0.00b	0.05 ± 0.02b	0.11 ± 0.01c

Values are means±SE. Different letters indicate significant differences at the 0.05 probability level.

**Table 2.** Chlorophyll *a*+*b* (chl(*a*+*b*)), the ratio between chlorophyll *a* and chlorophyll *b* (Chl*a*/*b*), nitrogen (N) content (%), maximum carboxylation rate (*V*_cmax_), and maximum electron transport rate (*J*_max_) based on the chloroplastic CO_2_ concentration (*C*_c_) and electron transport rate from chlorophyll fluorescence (*J*_flu_) calibrated by electron transport from gas exchange (*J*_A_) under 2% O_2_ conditions

	Chl(*a*+*b*) (mg dm^−2^)	Chl*a*/*b* (%)	N content (%)	*V*_cmax-*C*c_ (μmol m^−2^ s^−1^)	*J*_max-*C*c_ (μmol m^−2^ s^−1^)	*J*_flu_ (μmol m^−2^ s^−1^)
Leaf	5.73 ± 0.25a	3.14 ± 0.21a	3.62 ± 0.44a	526.7 ± 65.0a	456.0 ± 68.0a	345.4 ± 24.3a
Bract	2.15 ± 0.17b	2.51 ± 0.08b	2.86 ± 0.07b	39.3 ± 9.0b	52.0 ± 7.9b	55.0 ± 0.5b

Values are means±SE. Different letters indicate significant differences at the 0.05 probability level.

**Fig. 4.**
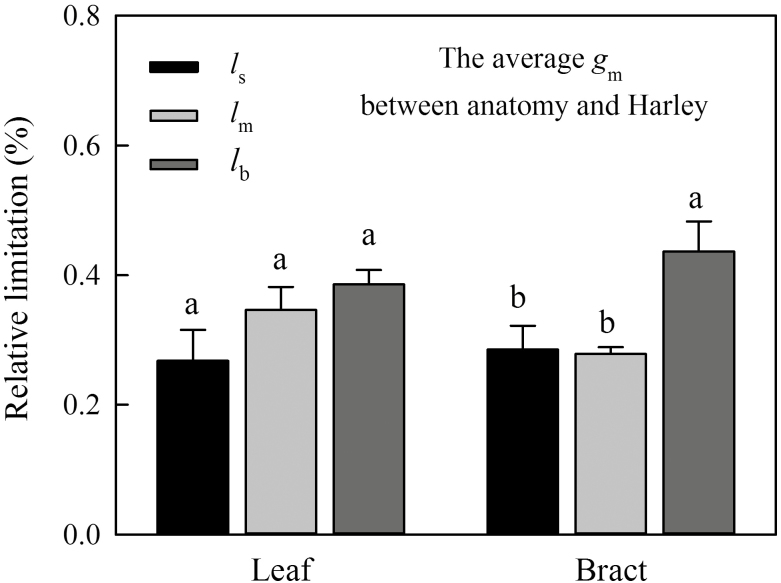
Relative limitation analysis of photosynthesis in the leaves and bracts of cotton under normal ambient conditions. The total relative photosynthetic limitation was composed of stomatal (*l*_s_), mesophyll conductance (*l*_m_), and biochemical limitation (*l*_b_). *l*_m_ was calculated using the average *g*_m_ between the anatomy and Harley methods. Values are means±SE. Different letters indicate significant differences between *l*_s_, *l*_m_, and *l*_b_ at the 0.05 probability level.

At the low values found in bracts, the accuracy of the estimates of *g*_m_ is low and the photosynthesis limitation analysis is very sensitive to small variation in any of its input parameters. Consequently, the results of the limitation analysis were completely different depending on which estimate we used (Fig. 4; Supplementary Fig. S2). Still, the Harley and the anatomy methods rely on completely independent assumptions (they have no single assumption in common), and yet both indicated low *g*_m_ in bracts (see the anatomy method results in the next section). Because of the aforementioned accuracy problems, the absolute values, however, have to be taken with caution. The ‘real’ values would very likely be somewhere in between the two extremes represented by the Harley method on the one hand and the anatomy-based estimates on the other. For this reason we used the average of both methods to run the photosynthesis limitation analysis (Fig. 4). There was no significant difference between *l*_m_ based on the average *g*_m_ between the anatomy and Harley methods and *l*_s_ in bracts (Fig. 4). However, *l*_b_ was higher than *l*_s_ and *l*_m_ in bracts (Fig. 4). Leaves had the same level of *l*_s_, *l*_m_, and *l*_b_ (Fig. 4).

### Anatomical measurements of cotton leaves and bracts

**Fig. 5.**
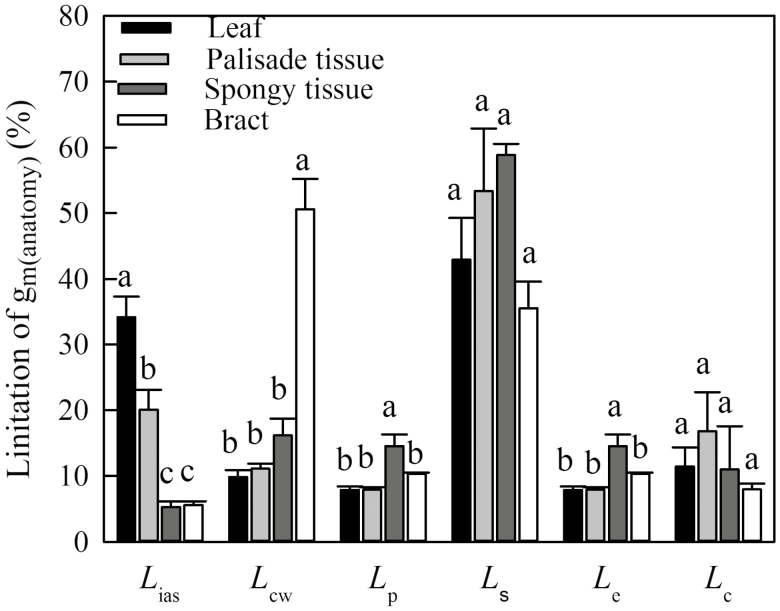
Quantitative analysis of partial limitation of mesophyll conductance modeled (*g*_m(anatomy)_) in the palisade and spongy tissue of leaves and the bracts. *L*_ias_, the limitation derived from the gas phase components; *L*_cw_, the limitation derived from the cell wall; *L*_p_, the limitation derived from the plasmalemma; *L*_s_, the limitation derived from the stroma; *L*_e_, the limitation derived from the chloroplast envelope; *L*_c_, the limitation derived from the cytoplast. Different letters indicate significant differences between palisade, spongy and bracts at the 0.05 probability level.

In the C_3_ cotton leaves, two types of chlorenchyma are found, palisade tissue and spongy tissue. In order to compare the structural differences between leaf and bract, palisade and spongy tissue of leaf and bract were quantified separately. In leaves, mesophyll tissue is differentiated into palisade and spongy mesophyll, the palisade tissue being more compact with a lower porosity (*f*_ias_; see Supplementary Fig. S3A, B). However, in the cotton bract, only one type of tissue was found, which was similar to the leaf spongy mesophyll (Fig. S3A, B). Although the LMA, leaf thickness (*T*) and density (*D*) in the cotton leaf were significantly higher than those in the bract (Table 3), we observed that there were no differences in the *S*_m_/*S*, *S*_c_/*S*, chloroplast thickness (*T*_chl_) and Area_chl_ between spongy tissue of leaves and bracts (Table 3). Bracts had higher *f*_ias_ and cell wall thickness (*T*_cw_) than spongy tissue of leaves (Table 3). The spongy tissue of leaves and bracts also showed similar anatomical structure (Fig. S3). Quantitative limitations of *g*_m_ modeled by anatomy were estimated according to the component diffusion conductance of the corresponding diffusion pathways (Fig. 5). The limitation derived from the gas phase components (*L*_ias_) (5–34%) was lower than the total limitation from liquid phase components. In the liquid phase, the palisade and spongy tissue of leaves revealed the highest limitation by the stroma (*L*_s_) of around 50%. There was no significant difference in the *L*_s_ between palisade and spongy tissue of leaves and bracts. In bracts, cell walls accounted for up to 50% of the limitations, compared with only 11% and 16% in palisade tissue and spongy tissue, respectively. The limitations derived from plasmalemma (*L*_p_) and chloroplast envelop (*L*_e_) in bracts were lower than those in spongy tissues, but were similar to those of palisade tissues of leaves.

**Table 3:** Leaf mass per unit area (LMA), leaf thickness (*T*), density (*D*), mesophyll thickness (*T*_mes_), the surface of mesophyll cells and chloroplasts exposed to leaf intercellular air spaces (*S*_m_/*S* and *S*_c_/*S*; µm^2^ µm^−2^), the chloroplast thickness, the thickness of the cytoplasm between the cell membrane and the chloroplast (*T*_cyt_), the cross-section area of chloroplast (Area_chl_), the volume fraction of intercellular air space (*f*_ias_) and cell wall thickness (*T*_cw_) in the cotton leaves and bracts

		LMA (g m^−2^)	*T* (μm)	*D* (g cm^−3^)	*T*_mes_ (μm)	*S*_m_/*S*	*S*_c_/*S*	*T*_chl_ (μm)	*T*_cyt_ (μm)	Area_chl_ (μm^2^)	*f*_ias_ (%)	*T*_cw_ (μm)
Leaf	Total	128.2 ± 0.5a	411 ± 10.2a	0.32 ± 0.00a	375 ± 6.6a	39.96 ± 2.3a	29.66 ± 2.5a	1.79 ± 0.28a	0.24 ± 0.07a	26.45 ± 4.1b	0.47 ± 0.02c	0.18 ± 0.01b
	Palisade tissue	**—**	**—**	**—**	195 ± 2.5c	28.9 ± 0.3b	22.6 ± 0.9b	2.22 ± 0.47a	0.34 ± 0.11a	37.81 ± 3.4a	0.33 ± 0.03d	0.20 ± 0.01b
	Spongy tissue	**—**	**—**	**—**	173 ± 4.2c	11.1 ± 2.1c	7.1 ± 0.9c	1.35 ± 0.15a	0.14 ± 0.09a	18.51 ± 2.1c	0.60 ± 0.01b	0.17 ± 0.01b
Bract	Total	53.1 ± 2.4b	350 ± 18.8b	0.15 ± 0.01b	300 ± 18.0b	14.6 ± 0.3c	7.4 ± 0.6c	1.12 ± 0.12a	0.13 ± 0.02a	15.65 ± 1.3c	0.75 ± 0.02a	0.45 ± 0.02a

Values were means±SE. Different letters indicate significant differences at the 0.05 probability level.

## Discussion

### Lower *A*_N_ in bracts than in leaves is due to co-limiting CO_2_ diffusion and biochemistry

It is well known that leaves are the main photosynthetic organs in plant species, but numerous researchers have shown that non-leaf green organs are also an important source of assimilated carbon (Tambussi *et al.*, 2007; Redondo-Gómez *et al.*, 2010; Pengelly *et al.*, 2011; Hu *et al.*, 2013; Jia *et al.*, 2015; Zhang *et al.*, 2015) and make a considerable contribution to terrestrial carbon exchange. In the case of cotton, bracts also have a photosynthetic function and contribute to carbon gain (Zhang *et al.*, 2010; Hu *et al.*, 2012, 2013). Moreover, it has been shown that some non-leaf green organs also have a strong stress tolerance, such as salt tolerance of rosette bracts (Redondo-Gómez *et al.*, 2010) and drought tolerance of cotton bracts (Zhang *et al.*, 2015) and wheat ears (Jia *et al.*, 2015). Hence, it is possible that, under abiotic stress conditions, non-leaf green organs make a considerable contribution to the carbon cycle.

Despite their importance, no previous study has focused on photosynthetic limitations and their anatomical basis in cotton bracts. In our study, *A*_N_ expressed on an area basis in bracts saturated at low irradiance (Fig. 2B; 3.58 μmol CO_2_ m^−2^ s^−1^), suggesting that light intensity was not the most important limiting factor for bract photosynthesis. It is likely that bracts can tolerate and thrive in low light intensity due to their growth in a shaded position over an evolutionary time of at least 1.1 million years since the appearance of tetraploid cotton (Hu *et al.*, 2013). A lower Chl*a*/*b* (Table 1) in the bract may be also a long-term adaption to capture more light. Björkman (1981) also suggested controlling the Chl*a*/*b* is one way to adapt the photosynthetic function to light.

The high values of photosynthesis observed in cotton leaves in this study (Table 1) were similar to those already reported for this species (Ephrath *et al.*, 1990; Faver and Gerik, 1996). Consequently, net CO_2_ assimilation rate (*A*_N_) values in cotton bracts were 90% lower than those obtained for leaves (Table 1; Fig. 2), which is accompanied by lower values of all the parameters related to photosynthesis. *g*_s_ in bracts was only about 10% that of leaves, *g*_m_ 8–36% (depending on which *g*_m_ estimate was used), *V*_cmax_ 7.5% and *J* 11–16% (depending on whether considering *J*_max_ or *J*_flu_). In this study, *g*_m_ was estimated by three independent methods that gave similar results for leaves, but this similarity was not found in bracts, with a significantly higher *g*_m(anatomy)_ than *g*_m(Harley)_ (Table 1). This may be partly due to the estimation biases of the currently available techniques. For instance, the variable *J* method was influenced by accuracy of *C*_i_ estimation (Gu and Sun, 2014) and (photo) respiratory CO_2_ recycling (Tholen *et al.*, 2012). We did our best to ensure the accuracy of *C*_i_ through calibration. Although the variable *J* method cannot rule out the effect of (photo) respiratory CO_2_ recycling, it is unlikely that this alone causes such a big difference in *g*_m(Harley)_ between leaf and bract and between *g*_m(anatomy)_ and *g*_m(Harley)_. In addition, the estimation of *g*_m(anatomy)_ is also subject to uncertainties. For instance, variable cell wall porosity was considered as a function of cell wall thickness (see ‘Materials and methods’ and Tosens *et al.*, 2016). But there was still a highly apparent discrepancy or inconsistency between *g*_m(anatomy)_ and *g*_m(Harley)_, and the difference in *g*_m(anatomy)_ between leaf and bract could not account for the observed difference in photosynthesis (Table 1). The overestimation of *g*_m(anatomy)_ in the bract could be because the anatomical model does not account for variations in some biochemical properties (e.g. the expression of aquaporins and carbonic anhydrase) that might be involved in the CO_2_ diffusion. In our experiment, a conservative constant value (0.0035 m s^−1^) was used to estimate the *g*_pl_ and *g*_en_ as suggested in Evans *et al.* (1994) and Tosens *et al.* (2012*a*), but membrane permeability is affected by the expression of aquaporins and varies among different organs, species, and environments. In order to test the role of membrane permeability, we tried different values that have been reported in the literature (see Table 1 in Evans *et al.*, 2009). When *g*_pl_ and *g*_en_ in the bract were replaced by 0.0008 m s^−1^ (which is the permeability reported for yeast cells), *g*_m(anatomy)_ was 0.069 mol CO_2_ m^−2^ s^−1^, i.e. much closer to *g*_m(Harley)_. Therefore, membrane permeability can be another potential cause of (i) the huge difference in photosynthesis between leaf and bract, and (ii) the discrepancy between *g*_m(anatomy)_ and *g*_m(Harley)_. This is why, considering that the actual value may be somewhere in between the extremes of the estimations, we have used the average *g*_m_ between anatomy and Harley values for the limitation analyses on photosynthesis. Both the similarity in the reductions of all the parameters related to photosynthesis (Table 1) and the relative limitation analysis (Fig. 4) confirmed that CO_2_ diffusion and biochemistry co-limit bract photosynthesis in a similar way. High biochemical limitation in bracts could be caused by a low Rubisco activity, as Bota *et al.* (2004) proved its good agreement with *V*_cmax_ derived from *A*_N_–*C*_c_ curves. Bracts had very low *g*_s_, and high stomatal limitation would be likely due to the limited hydraulic capacity caused by the low main vein density.

### Subcellular anatomical traits play important roles in setting *g*_m_ of bracts

Leaf mass per unit area (LMA) is an integrative trait of leaf structural characteristics affecting *g*_m_. It is mainly dependent on leaf thickness and density (Niinemets, 2015). John *et al.* (2017) reported that the number of cell layers and cell volume that is associated with leaf thickness and density are among the most important intrinsic drivers of LMA. Because leaf thickness and density are closely related to the *S*_c_/*S* and *T*_cw_, theoretically LMA has an important role in setting *g*_m_. Leaf thickness was 1.25 times larger than bract thickness and leaf density was 1.89 times larger than bract density (Table 3). These results suggest that higher density in the leaf mainly contributed to larger LMA. A lower proportion of mesophyll and a higher *f*_ias_ due to random cell arrangement and lower cell numbers led to lower density in bracts (Table 3; Supplementary Fig. S3). While early studies have shown that there is a negative relationship between LMA and *g*_m_ across broad functional groups and within species (Flexas *et al.*, 2008; Niinemets *et al.*, 2009; Galmés *et al.*, 2011; Tosens *et al.*, 2016), this is not consistent with our results, which show that bracts have lower LMA than leaves despite having a higher *T*_cw_ (Table 3). Recently, Onoda *et al.* (2017) highlighted that subcellular anatomical traits such as *T*_cw_, *S*_m_/*S*, and *S*_c_/*S* are much more important than LMA in setting *g*_m_. Similarly, Peguero-Pina *et al.* (2017) showed that these parameters as well as *f*_ias_ can mask the effects of LMA on *g*_m_.

CO_2_ diffuses from the intercellular air spaces to the sites of carboxylation within chloroplasts in gas phases largely affected by leaf porosity, reflected by *f*_ias_ (Hanba *et al*., 1999), and liquid phases largely affected by *T*_cw_, *S*_m_/*S*, and *S*_c_/*S* (Evans *et al.*, 1994; Hanba *et al.*, 2004; Terashima *et al.*, 2011; Tomás *et al.*, 2013; Peguero-Pina *et al.*, 2016). Evans *et al.* (1994) concluded that *g*_ias_ is so large that it is not a major determinant of *g*_m_ in leaves. Instead, *T*_cw_, *S*_m_/*S*, and *S*_c_/*S*, which affect *g*_liq_, are considered the main determinants of differences in *g*_m_ among species (Terashima *et al.*, 2011; Tomás *et al.*, 2013; Peguero-Pina *et al.*, 2016, 2017). Bracts with thin mesophyll thickness had smaller *S*_m_/*S* and *S*_c_/*S* than leaves (Table 2). Several studies have indicated larger *S*_m_/*S* and *S*_c_/*S* in thicker leaves (Hanba *et al*., 1999; Terashima *et al.*, 2006; Peguero-Pina *et al.*, 2016), likely reflecting the more developed palisade tissues in the thicker leaves. The smaller *S*_m_/*S* and *S*_c_/*S* were also likely due to higher *f*_ias_ that was caused by the fewer and smaller cells in bracts. Based on this, we quantified separately the anatomical structure of palisade and spongy tissues in leaves (Table 3). The quantitative results indicated that *S*_m_/*S* and *S*_c_/*S* of bracts were similar to the spongy tissue of leaves, which contributed to lower *g*_m_ in bracts and the spongy tissue of the leaves. In addition, chloroplast size and thickness are also an important factor limiting CO_2_ diffusion to Rubisco (Tomás *et al.*, 2013; Tosens *et al.*, 2016; Veromann-Jürgenson *et al.*, 2017), and thus smaller Area_chl_ in bracts and the spongy tissue of the leaves was also a cause of lower *g*_m_. Although numerous studies reported that *T*_cw_ is generally higher in woody species with thick leaves (Evans *et al.*, 2009; Terashima *et al.*, 2011; Tosens *et al.*, 2012b;Veromann-Jürgenson *et al.*, 2017), higher *T*_cw_ was observed in bracts than in either the palisade or spongy tissues of leaves. This is possibly due to a larger nitrogen investment in structural construction (i.e. the cell wall construction) in bracts than in leaves, which is supported by lower *A*_N-N_ expressed on the basis of nitrogen content in bracts (Fig. 3C). In this sense, relative to leaves, a larger nitrogen investment in cell wall construction led to lower *g*_m_ in bracts. Instead, mesophyll conductance of bracts was similar to that of spongy tissues, the highest values being those of palisade tissue. The further anatomical limitation analysis (Fig. 5) showed that the limitation derived from the cell wall (*L*_cw_) is higher in bracts than in spongy tissue, but the cumulative effects of subtle differences in each subcellular structure, especially the chloroplast traits, compensate *L*_cw_ to yield similar *g*_m_ in bracts and spongy tissue. However, there was smaller *g*_m_ in bracts than in palisade tissue because of the larger *T*_cw_, greater *f*_ias_, smaller chloroplasts, and lower *S*_m_/*S* and *S*_c_/*S*.

### Conclusion

In summary, net CO_2_ assimilation rate (*A*_N_) was lower in cotton bracts than in leaves, and this was due to concomitant and similar limitations by biochemistry, stomatal conductance (*g*_s_), and mesophyll diffusion conductance (*g*_m_). Concerning *g*_m_, we provide the first report showing that anatomical traits setting the limits for *g*_m_ in leaves also operate in non-leaf photosynthetic tissues like bracts. Specifically, larger *T*_cw_ and *f*_ias_, smaller and fewer chloroplasts, lower *S*_m_/*S* and *S*_c_/*S*, and, perhaps, smaller membrane permeability in bracts than in leaves led to lower *g*_m_. It has been shown that in leaves of angiosperm species (Flexas, 2016), but especially in crops (Nadal and Flexas, submitted), stomatal, mesophyll conductance and biochemical limitations to photosynthesis are of similar magnitude, for which significantly improving leaf photosynthetic capacity in crops cannot be achieved unless all three factors are improved (Flexas, 2016). Here we show that this might be similar in bracts. Since bracts contribute significantly to the photosynthetic carbon gain of plants (Zhang *et al.*, 2010, Hu *et al.*, 2012, 2013), the present results should be considered in future attempts to improve crop productivity by means of manipulating photosynthesis.

## Supplementary data

Supplementary data are available at JXB online.

Fig. S1. Daily maximum and minimum air temperature and precipitation during the growing season at the experimental field.

Fig. S2. Relative limitation analysis of photosynthesis for leaves and bracts of cotton under ambient conditions.

Fig. S3. Light and electron microscopy images of cotton leaves and bracts.

Supplementary Figure S1-S3
