# Supplementary material for: Mesophyll conductance in cotton bracts: anatomically determined internal CO2 diffusion constraints on photosynthesis
Source: J Exp Bot. 2018 Aug 14;69(22):5433–43. doi: 10.1093/jxb/ery296 (PMC6255706; doi:10.1093/jxb/ery296)
Supplement: Supplementary Figure S1-S3 [file ery296_suppl_supplementary_figures_s1-s3.pdf]

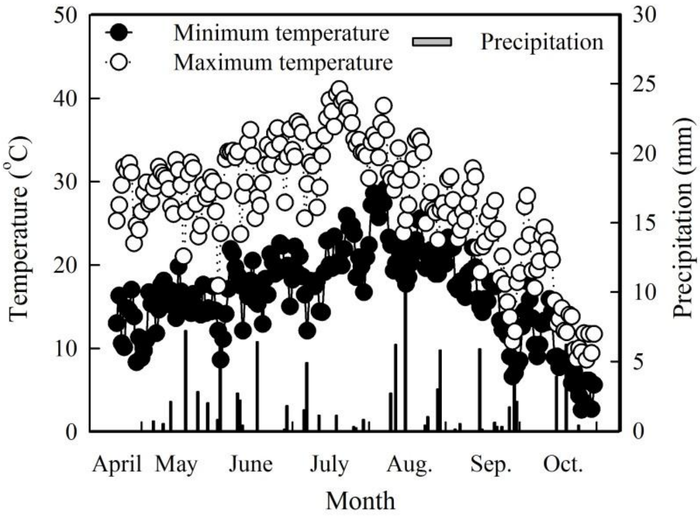

Fig. S1. Daily maximum and minimum air temperature (open and closed circles, respectively) and precipitation (bars) during the growing season at the experimental field.

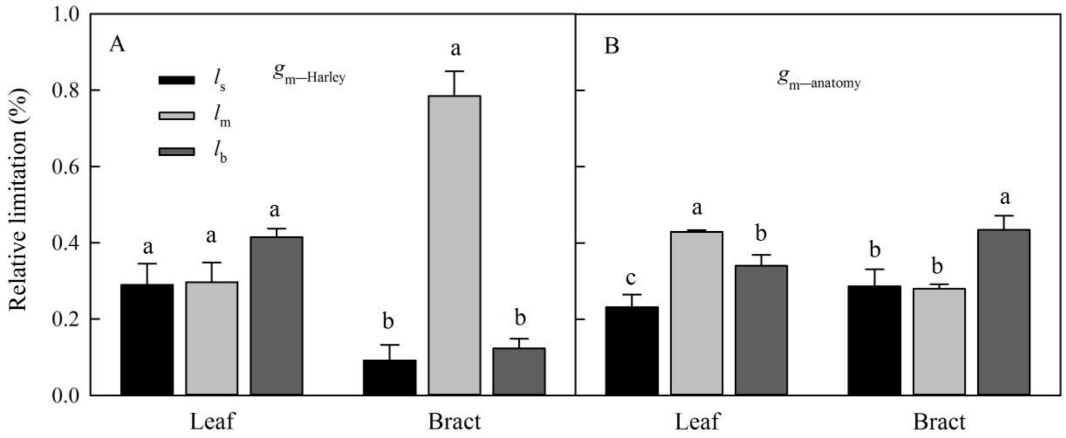

Fig. S2. Relative limitation analysis of photosynthesis for leaves and bracts of cotton under ambient conditions. The total relative photosynthetic limitation was composed of stomatal (l_s), mesophyll conductance (l_m), and biochemical limitation (l_b). A: l_m calculated using g_{m_Harley}; B: l_m calculated using $g_{m\_anatomy}$ instead of g_{m_Harley}. Values are means of three replicates ±standard error. Different letters indicate significant differences between l_s, l_m,and l_b at the 0.05 probability level.

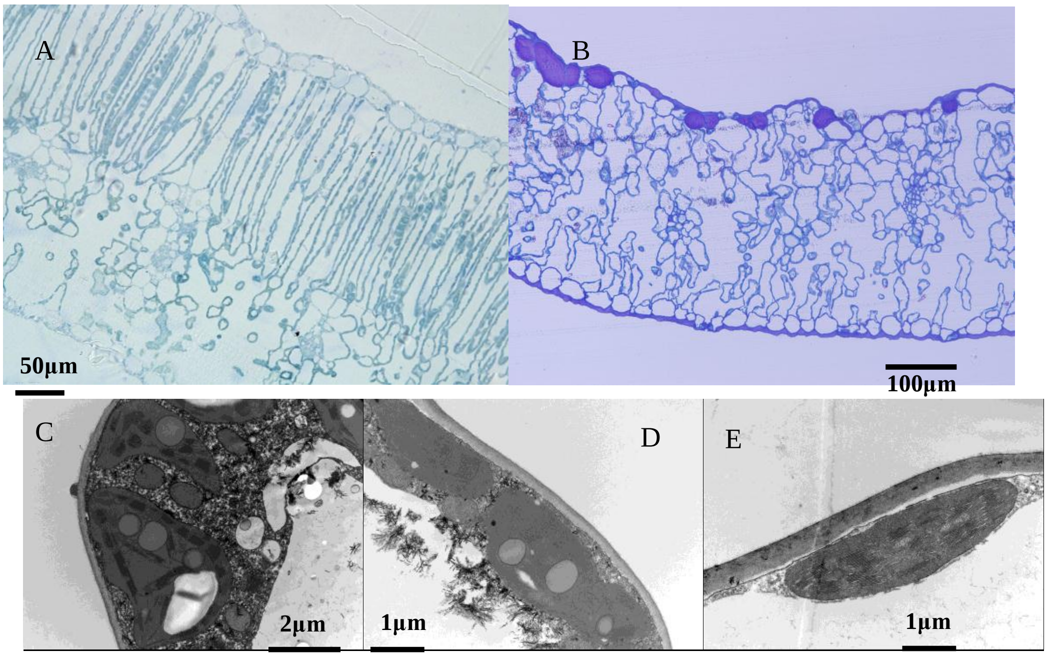

Fig. S3. Light and electron microscopy images of cotton leaves and bracts. A and B: light microscopy images of leaves and bracts, respectively; C, D and E: electron microscopy images of chloroplasts and cell walls for leaf palisade tissue (20000×), leaf spongy tissue (30000×) and bracts (30000×), respectively.
